# Consumption of Ultraprocessed Foods in a Sample of Adolescents With Obesity and Its Association With the Food Educational Style of Their Parent: Observational Study

**DOI:** 10.2196/28608

**Published:** 2021-11-15

**Authors:** Sylvie Borloz, Sophie Bucher Della Torre, Tinh-Hai Collet, Corinne Jotterand Chaparro

**Affiliations:** 1 Pediatric Service Department Woman-Mother-Child Lausanne University Hospital (CHUV) Lausanne Switzerland; 2 Department of Nutrition and Dietetics Geneva School of Health Sciences HES-SO University of Applied Sciences and Arts Western Switzerland Geneva Switzerland; 3 Service of Endocrinology, Diabetology, Nutrition and Therapeutic Education Geneva University Hospitals (HUG) Geneva Switzerland

**Keywords:** adolescent, obesity, ultraprocessed foods, qualitative food intake, food educational style, smartphone application

## Abstract

**Background:**

Both parental education and the food environment influence dietary intake and may therefore contribute to childhood obesity.

**Objective:**

We aimed to assess the consumption of ultraprocessed foods (UPFs) in a convenience sample of adolescents with obesity and to determine its association with the food educational style of their parent.

**Methods:**

This observational study included 24 participants, 12 adolescents (8 boys and 4 girls) aged from 12 to 14 years and their 12 parents, who were followed in a specialized pediatric obesity clinic in the French-speaking part of Switzerland. The adolescents were asked to take a photograph with a smartphone application of all meals and beverages consumed in their daily routine over 14 consecutive days. They evaluated their parent’s food educational style using the Kids’ Child Feeding Questionnaire. The parent who was present at the study visits also completed the Feeding Style Questionnaire. A dietitian analyzed the pictures to extract food group portions and to identify UPFs using the NOVA classification. A nonparametric statistical test was used to investigate associations between UPF intake and food educational style.

**Results:**

Overall, the adolescents had unbalanced dietary habits compared to national recommendations. They consumed an insufficient quantity of vegetables, fruits, dairy products, and starchy foods and an excessive amount of meat portions and sugary and fatty products compared to the current Swiss recommendations. Their consumption of UPFs accounted for 20% of their food intake. All adolescents defined their parent as being restrictive in terms of diet, with a mean parental restriction score of 3.3±SD 0.4 (norm median=2.1). No parent reported a permissive food educational style. A higher intake of UPFs was associated with a lower parental restriction score (*P*=.04).

**Conclusions:**

Despite being followed in a specialized pediatric obesity clinic, this small group of adolescents had an unbalanced diet, which included 20% UPFs. The intake of UPFs was lower in participants whose parent was more restrictive, suggesting the importance of parents as role models and to provide adequate food at home.

**Trial Registration:**

ClinicalTrials.gov NCT03241121; https://clinicaltrials.gov/ct2/show/NCT03241121

## Introduction

Childhood obesity is a significant public health challenge, with an increasing prevalence worldwide and multiple long-lasting consequences [1]. Its causes are multiple, with the environment and behaviors interacting with the individual genetic background [2]. Excessive consumption of calorie-dense foods containing high levels of saturated fats, trans-fatty acids, free sugars, or salt contribute to obesity and diabetes, as well as other noncommunicable diseases [3-5].

In the past decades, the level of food processing has significantly increased [6]. Recent studies in adults and children have suggested an association between the consumption of ultraprocessed foods (UPFs) and an increased risk of being overweight or obese and having metabolic disorders [6-9]. A systematic review found that UPF consumption was positively associated with body fat during childhood and adolescence in 14 of the 26 included studies [7]. The authors concluded that there is a need to use a standardized classification that considers the level of food processing to promote comparability between studies, such as the recent food NOVA classification. The NOVA classification divides food items into four groups according to their degree of processing: (1) low or unprocessed foods, (2) culinary ingredients, (3) processed foods, and (4) UPFs [9]. UPFs are industrial products that not only contain fat, sugar, and salt but also include additives or ingredients not normally used in home food preparation, such as hydrogenated or unesterified oils, protein isolate, maltodextrin, casein, and gluten [10,11]. One study showed that Swedish children increased their UPF consumption by 142% from 1960 to 2010 [9]. UPF consumption accounted for 25%-60% of the total daily energy intake in adults of 19 European countries [12]. Currently, experts recommend limiting UPF consumption, even though no recommendation has yet been determined for the maximal amount or frequency [6].

Both the education and the environment influence dietary intake in general, and in addition, in children, parental education and the food environment provided are crucial. Ellyn Satter [13] described a model of the division of responsibilities between children and parents. Fundamental to the parental tasks is trusting children to determine *how much* and whether to eat from *what* parents provide. This model is complementary to the concept of the food educational style, developed by Johnson and Birch in 1994 [14], demonstrating that a high degree of parental control over a child’s intake is associated with decreased dietary regulation and a higher weight of the child. The so-called authoritative parenting style is associated with a favorable food environment, as opposed to the permissive or authoritarian style [15]. Based on a model published in 2017, a child’s eating behavior and the parents' food educational style could explain the onset of obesity in 19% of the cases [16]. A study published in 2019 showed a positive effect of healthy parental eating practices and the authoritative food educational style on the food habits of 13-year-old adolescents who were overweight or obese [17].

In this observational study, we aimed to assess the consumption of UPFs in a group of Swiss adolescents with obesity and to determine its association with the food educational style of their parent.

## Methods

### Setting and Participants

This observational study included adolescents aged 12-14 years who were followed in a specialized pediatric obesity clinic at the Lausanne University Hospital, Lausanne, Switzerland, and one of their parents. The study was an observational nested study of the SwissChronoFood trial [18] (Clinicaltrials.gov registration no NCT03241121). The protocol was approved by the ethics committee of the Canton of Vaud, Lausanne, Switzerland. Each adolescent participant and their parent were informed of the study details and signed written consent.

**Figure 1:**
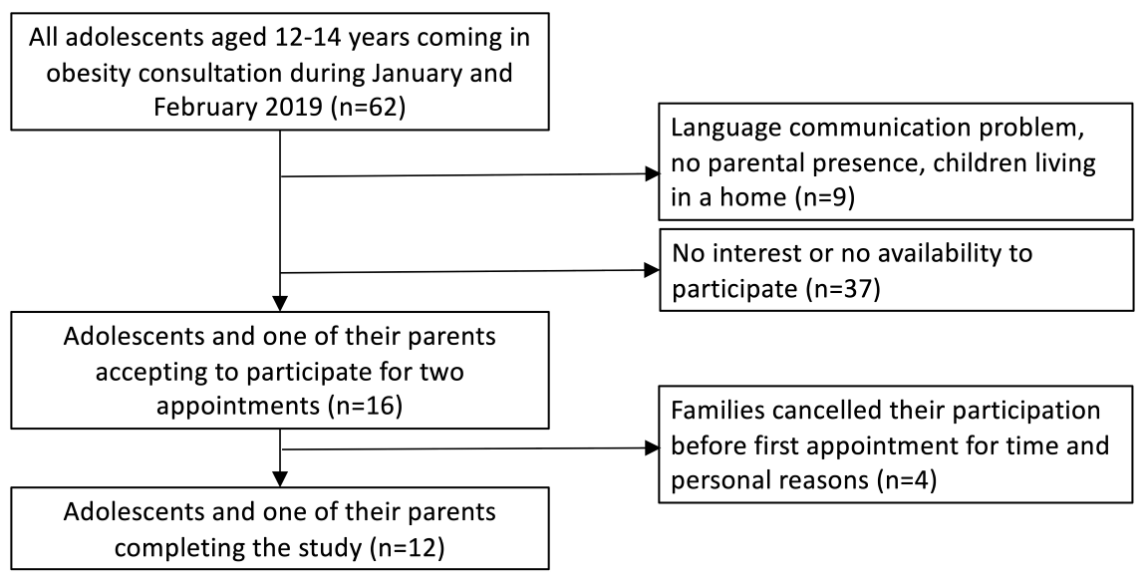
Recruitment process of adolescents and one of their parents.

The families were sent to the pediatric obesity clinic by their pediatrician. At the time of inclusion, the senior dietician (author SB) had followed the adolescents for several months. She invited all adolescents aged 12-14 years who had an appointment at the clinic from January to February 2019 to participate in the study. Of the 62 adolescents aged 12-14 years informed about the study, 37 declined because of a lack of interest or time to attend the study visits and 9 because of a language barrier or the lack of a parent available to attend the study visits (Figure 1). Of the 16 adolescents and their respective parent who agreed to take part in the study, 4 families had to cancel their participation before inclusion, thus leading to a final sample size of 12 adolescents and 12 parents. The nutritional intake of the adolescents was assessed over a 2-week period, including 2 face-to-face visits with a senior dietician (SB) and a phone meeting in the interval, between January and March 2019.

Demographic and anthropometric data were collected in the first visit. After 1 week, the dietician had a phone meeting with the adolescents to question them about the use of the smartphone application (explained later) and to encourage them to continue taking pictures conscientiously. At the last visit, the dietitian checked the pictures collected by the smartphone application and performed a 24-hour food recall.

The z score of the body mass index (BMI) according to age was used to define overweight and obesity. According to the World Health Organization [19], overweight is defined as a BMI z score of >1, obesity as a BMI z score of >2, and extreme obesity as a BMI z score of >3. For parents, the adult categories of overweight (BMI=25-30 kg/m^2^) and obesity (BMI>30 kg/m^2^) were used [1].

### Assessment of Food Intake

All adolescents used a smartphone application to take pictures of all consumed food and beverages, except water, over 14 consecutive days. They could annotate each picture with a text description. We compared the food pictures collected by the food application and the 24-hour food recall performed at the second visit. The senior dietician (SB) manually counted the number of food portions consumed each day by each adolescent and estimated the number of servings from each picture. Food items were grouped according to the Swiss food pyramid [19] as follows: fruit, vegetables, starchy food, meat/fish/egg/tofu, dairy products, sugary products, fatty food, and sugar-sweetened beverages. The intake of cooking fats, sauces, and salad dressings was not analyzed, as these could not be accurately assessed from the pictures collected and the text annotations. The frequency of consumption of each food group was then compared to the Swiss Nutrition Society (SNS) recommendations [20]. Finally, UPFs were identified from food pictures and the 24-hour food recall, according to the NOVA classification [10].

### Assessment of the Parental Food Education Style

The parental food education style was assessed from the perspectives of both the adolescents and their parent. At the first visit, the adolescents completed the Kids' Child Feeding Questionnaire in a separate room from the accompanying parent [14]. This questionnaire explores an adolescent's perspective of two dimensions, parental pressure and parental restriction on their feeding, and has been validated in French [21]. The scale ranges from 0 to 4: 0 meaning no pressure and no restriction and 4 meaning maximal pressure and maximal restriction. Our results with the Kids' Child Feeding Questionnaire were compared with the median scores of 2.1 for restriction and 1.99 for pressure, which were obtained in a French pediatric population that we considered as a norm [21].

Although the adolescents were completing the questionnaire in a separate room, the parents answered the Feeding Style Questionnaire, which explores a parent’s perspective in eight problematic situations (eg, your child wants to eat pasta, when you intended to cook vegetables) and is also validated in French [22]. This questionnaire assesses three dimensions, described as authoritarian, authoritative, and permissive [16]. In short, the *authoritarian style* includes strict rules given by parents without discussion, the *authoritative style* is a more democratic style with rules and a discussion of these rules, and the *permissive style* has few or no rules, thus following the wishes of the adolescent more. Each dimension received a score on a 4-point scale from very unlikely to very likely. The dimension with the highest score determined the dominant feeding style of each parent.

### Statistical Analysis

Data are reported as the mean±SD, unless stated otherwise. Nonparametric tests were used due to the small sample size. We compared the rank-sum test between UPF intake and food educational style (restriction, pressure to eat, and authoritarian, authoritative, and permissive dimensions) with the Wilcoxon-Mann-Whitney test. For analysis of the perceived parental dietary restriction, we defined groups of low restriction and high restriction using the median value of 3.25. *P*<.05 was considered statistically significant. The Stata 15 software package (College Station, TX, USA) was used. No missing data were found for the variables of interest.

## Results

### Characteristics of the Participants

We included 12 adolescents, 8 boys and 4 girls, aged 12-14 years and 12 parents, 8 mothers and 4 fathers, aged 37-55 years. At the time of the study, the adolescents had been followed in the specialized pediatric obesity clinic for several months. Of the 12 adolescents, 11 (91.6%) were obese and 1 (8.4%) had lost weight, thus changing from the obese category to the overweight category. Most parents were overweight or obese (n=11), and 10 (83.3%) worked at an activity level of ≥70%, except for 2 (16.7%) parents on disability insurance. Five of the included parents (42%) were separated, but the adolescents spent almost all of their time with the parent who was present at the study visits. Table 1 shows the characteristics of adolescents and parents.

**Table 1:** Characteristics of adolescents and parents.

Characteristics		Value
**Adolescents’ characteristics**		
	Number	12 (4 girls/8 boys)
	Age (years)	13.3±0.6 (12.0-14.3)^b^
	BMI^a^ (kg/m^2^)	30.0±2.6 (24.9-33.7)^b^
	BMI (z score)^c^	2.7±0.4 (1.9-3.4)^b^
**Parents’ characteristics**		
	Number	12 (8 mothers/4 fathers)
	Age (years)	45.3±4.6 (37.0-55.0)^b^
	BMI (kg/m^2^)	29.1±3.2 (23.2-35.8)^b^
	Married or in a relationship with the other parent (%)	58.3
	Time spent with child (%)	97.9±7.0 (75-100)^b^
	Training after compulsory school (years)	3.2±2.9 (0-7)^b^
	Professional activity rate (%)	70.0±30.0 (0-100)^b^

^a^BMI: body mass index.

^b^Data are presented as the mean±SD (minimum-maximum range), unless stated otherwise.

^c^Obesity in adolescents was defined as a z score of BMI>2.

### Food Intake

Overall, the adolescents had unbalanced dietary habits compared to national recommendations (Table 2). Their consumption of fruit, vegetables, dairy products, and starchy foods was below the recommended frequencies for adolescents [20], while the consumption of the meat/fish/egg/tofu group, fatty products, and sugary products was above the recommendations [20]. The number of meals was close to 3 meals per day (2.8±0.5), although 5 adolescents skipped breakfast. A mean 1.6±0.6 portions of UPFs were consumed each day, representing 20% of the food portions consumed.

**Table 2:** Comparison of food consumption with the Swiss recommended daily portions [20].

Food groups	Number of portions per day (mean±SD)	Swiss national recommendations for 13-14-year-old adolescents (n)
Fruit^a^	0.4±0.3	2
Vegetables	1.2±0.6	3
Starchy foods	2.5±0.8	4.5
Meat, fish, egg, tofu	1.4±0.4	1
Dairy products^b^	1.1±0.3	3
Sugary products^c^	1.2±0.7	1
Fatty products^d^	1.3±0.7	1
Sweet beverages	0.2±0.3	0
UPF^e^ intake	1.6±0.6	—^f^
UPF portions/total number of food portions (%)	20.9±3.6	—

^a^Including a maximum of 1 glass of fruit juice per day and a maximum of 1 fruit compote per day.

^b^Including milk, yogurt, cheese, and milk drinks.

^c^Including jam, honey, chocolate, cookies, cakes, fruit yogurt, candies, sodas, ketchup, sweet sauce for nems.

^d^Including sausages, crisps, breaded meat, chocolate, cookies, raclette, fondue, fat-containing sauces (carbonara, mayonnaise), lasagna, and pizza.

^e^UPF: ultraprocessed food (includes industrial prepackaged snacks, sweets, commercial biscuits, chips, sausages, ham, sodas, filled croissants, ravioli, tortellinis, spätzlis, fajitas, ketchup, mayonnaise, sweet and sour sauce, nems, milk drinks [eg, Danao^®^, Actimel^®^], toasted bread, pizza, dessert cream, and chocolate spread).

^f^No Swiss recommendations for UPF food group

### Food Educational Styles

**Figure 2:**
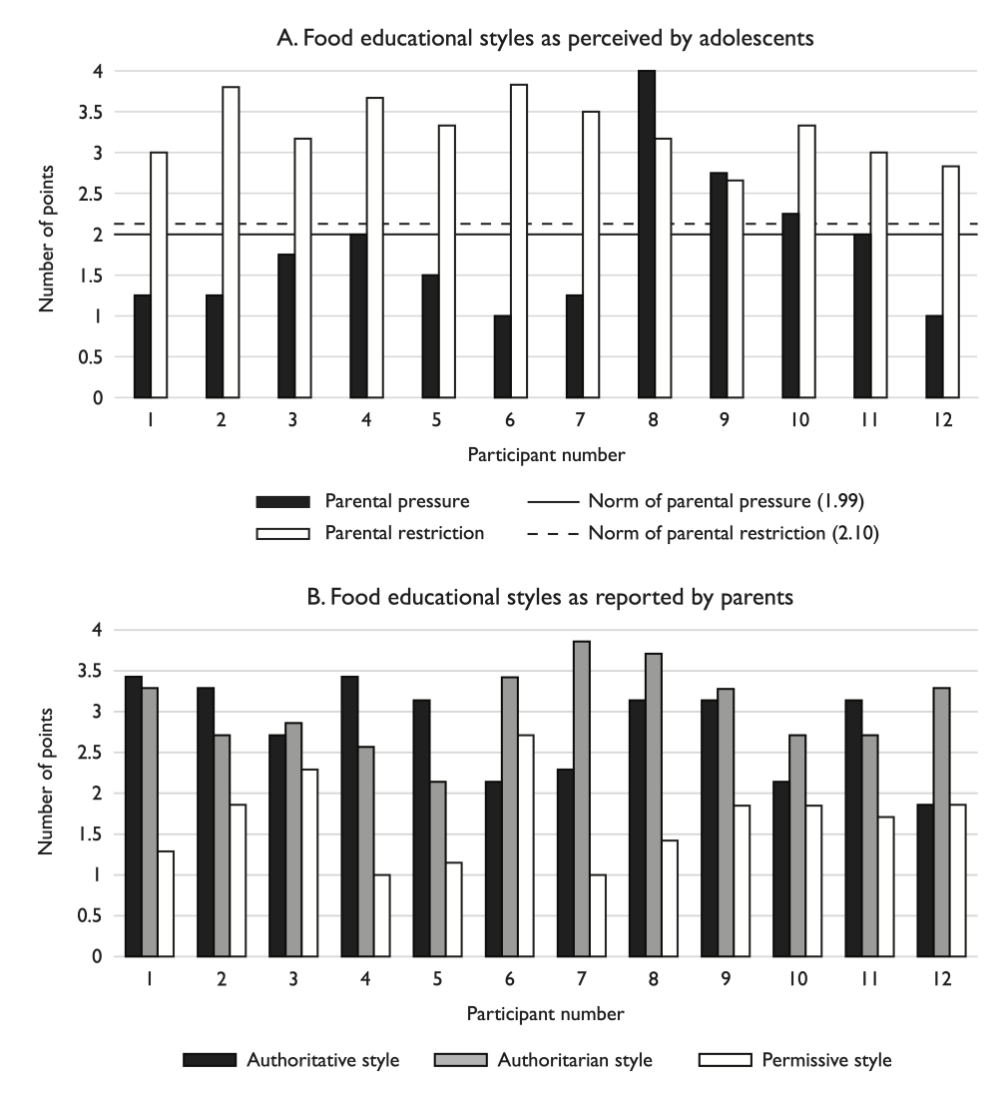
(A) Food educational styles perceived by the adolescents. Results of the Kids’ Child Feeding Questionnaire [20] completed by the adolescents. (B) Food educational styles reported by the parents, measured by the Feeding Style Questionnaire [21] completed by the parents.

According to the Kids’ Child Feeding Questionnaire [21] completed by the 12 adolescents, the mean parental restriction score was 3.27±0.37 (Figure 2A) and the mean parental pressure score was 1.83±0.87 (norm=1.99). For seven adolescents, the perceived parental pressure to eat was below the norm.

The Feeding Style Questionnaire completed by the 12 parents showed that the most common dietary educational style was the authoritative style, with a mean score of 3.05±0.51, followed by the authoritarian style (2.82±0.57). The permissive style had the lowest score (1.67±0.52). The authoritative style was predominant in seven parents, and the authoritarian style was predominant in five parents (Figure 2B). The permissive style was not dominant in any parent.

### Association Between UPF Consumption and Parents’ Food Educational Styles

**Figure 3:**
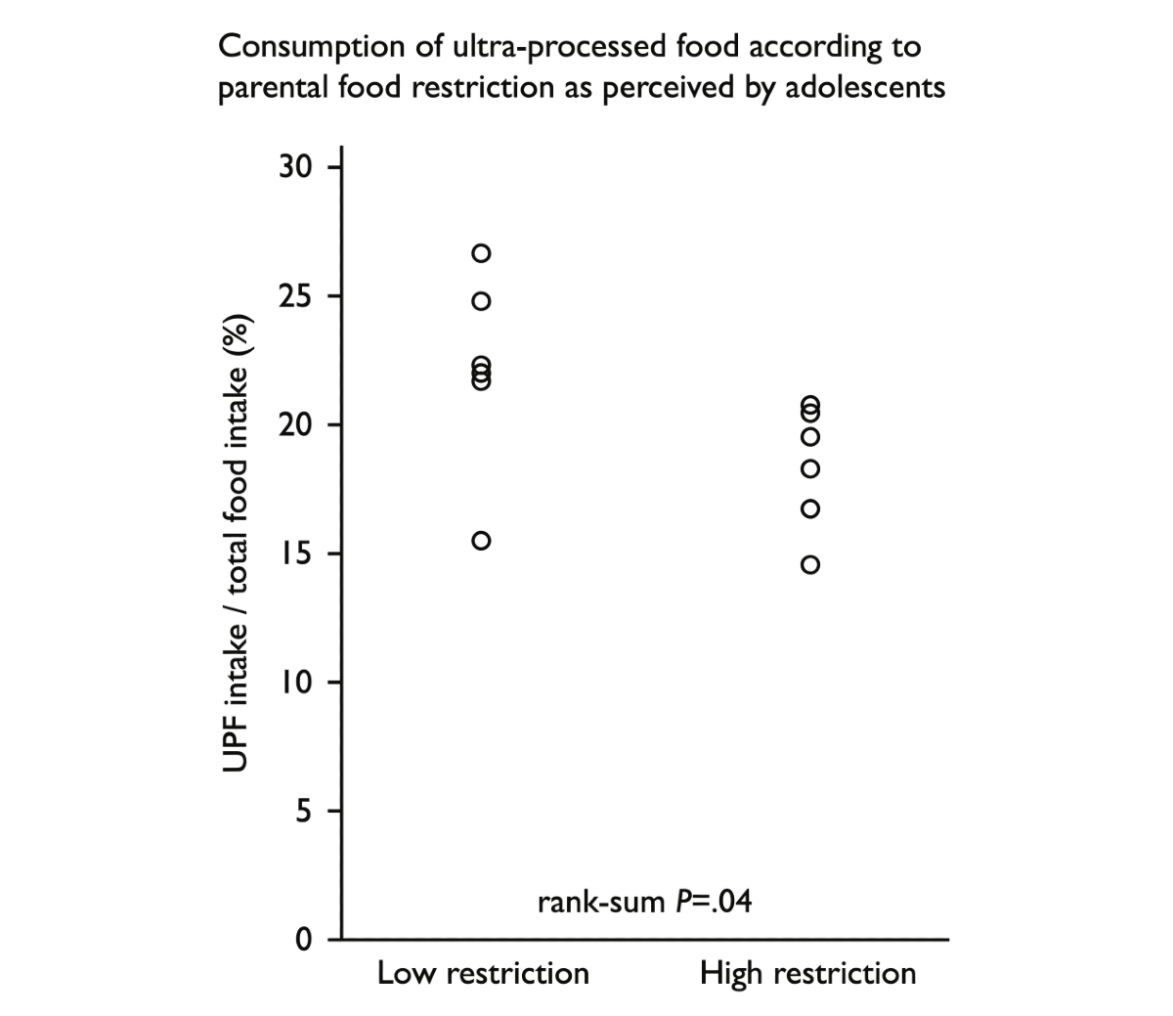
Consumption of UPFs according to the parental dietary restriction perceived by the adolescents. Association between the proportion of UPF intake out of the total food intake and the level of parental dietary restriction (rank-sum *P*=.04). UPF: ultraprocessed food.

When analyzing the adolescents’ dietary intake and the respective parent’s food educational styles, we found a significant association between the proportion of UPF intake compared to the total food intake and the level of parental dietary restriction (Figure 3).

## Discussion

### Principal Results

In this observational study conducted in the French-speaking part of Switzerland, the small group of adolescents with long-standing obesity had unbalanced eating habits, including excessive UPF consumption, despite being followed in a specialized pediatric obesity clinic. The adolescents perceived their parent as more restrictive than the norm, and none of the parents had a permissive food educational style. Lower UPF consumption was associated with a higher parental dietary restriction.

The reported diet was unbalanced, including 0.4 portions of fruit per day instead of the 2 portions recommended by the Swiss national recommendations [19], 1.2 portions of vegetables per day instead of 3, and 1.1 portions of dairy products instead of 3. To put this in perspective, these results are similar to those found in the Swiss adult general population, who also have self-reported intakes below the national recommendations [23].

In this study, UPF consumption was high, with 1.6 portions consumed per day, representing 20% of the foods consumed.

### Comparison With Prior Work

The comparison of these findings with other studies is limited, as UPF consumption is often reported as a percentage of daily energy intake and not in terms of portions per day. A study in adults found that UPFs reached an average 26% of daily energy intake, ranging from 10% to 50%, depending on the 19 European countries assessed [12]. A Brazilian study in school-age children observed that 48% of daily energy intake was provided by UPF consumption [7]. UPF consumption shows an upward trend across multiple countries and cultures, as seen in Swedish children who increased their UPF consumption by 142% between 1960 and 2010 [9]. In a large prospective cohort of French adults, UPF consumption was associated with increased weight gain [24].

UPFs contribute to an unbalanced diet due to their low nutritional quality, including a high content of added sugars, fats, or additives and a low content of fiber. The lack of prospective studies precludes a definitive conclusion on the causal relationship between UPF consumption and obesity [25]. However, observational studies have shown an association between UPF consumption and overweight, obesity, or metabolic disorders [6,24,25]. Therefore, many experts, such as the Canadian government [26], have called for a limit of their intake, without providing precise quantified recommendations [26]. One suggestion to reduce UPF consumption in children and adolescents is to develop parents' skills in identifying UPFs and provide them with practical tips on how to limit the UPFs’ frequency, reduce their portions, or replace them with raw foods. Moreover, parental food practices influence child practices [27,28]. A European survey in eight countries observed that the poor example of parents was a predictor of children's eating habits [28]. Similarly, a recent systematic review showed that parents’ own food consumption behavior and food availability at home are factors with the strongest association with food consumption of adolescents in the same household [28]. Another systematic review concluded that the availability of unhealthy foods at home is positively associated with snack intake [29]. Thus, a global family approach is necessary.

A permissive food educational style is recognized as promoting obesity [30]. In our study, all adolescents perceived their parent as highly restrictive in terms of diet, and the Feeding Style Questionnaire completed by the parents showed that the permissive educational style was the least common. The restrictive food educational style experienced by adolescents may be explained by the fact that parents wish to control their children’s excess weight by using dietary restriction. Several studies have shown that parental restriction is more frustrating than parental pressure and is associated with increased weight in children and adolescents with normal weight or who are overweight [21,30,31]. However, some degree of restriction may be beneficial to limit UPF intake. Interestingly, in this study, we found that adolescents who perceived a higher dietary restriction from their parent consumed significantly less UPFs. In addition, the consumption of sweet beverages was low (0.2 portions per day instead of the 2.4 portions in the Swiss adult population [23]) and could be explained by the fact that parents limited their access, as this is part of the advice given in the follow-up at the obesity clinic.

### Limitations

The main limitation of this study was the limited sample size, which included 12 adolescents and 12 parents. We contacted 62 adolescents followed in our pediatric obesity clinic and their parent to participate in the study. A total of 50 refused to participate, 37 due to a lack of interest or time and 9 due to a language barrier or the lack of a parent available to attend study visits; in addition, 4 families had to cancel their participation before the first visit. This shows the difficulty of recruiting this population in dietary studies involving longitudinal data collection. This could have led to a type I error, but our results are mostly exploratory and will help future studies in the form of preliminary results for sample size calculation and new hypotheses generation. Other limitations were the low response rate and the potential social desirability of participants who would only take pictures of the food they wished to show. Although the long duration of the data collection period provided detailed information about dietary habits and was a strength of this study, it might also be a limitation. Indeed, 2 weeks might have been too long for adolescents, leading to potential missing data, as shown by the comparison with the 24-hour food recall. The 24-hour food recall showed the consumption of more foods, such as highly processed foods, which accounted for 26% of the foods in the recall instead of 20% with the smartphone application. The data were collected between January and March, which might have affected the availability of fresh products. However, the availability and price of fresh products in Switzerland do not differ widely between seasons. Finally, the studied adolescents were followed in a specialized pediatric obesity clinic in the French-speaking part of Switzerland; thus, our findings may not be applicable to adolescent populations in other parts of the world or followed in other clinical settings. The main strengths of the study were the review of UPF consumption by a senior dietitian, which allowed an estimation of the number of UPFs compared to other foods; the use of a smartphone application to take food pictures; and the assessment of parental feeding practices, perceived by both the adolescents themselves and one of their parents. Our study relied on a smartphone application to collect data on eating behavior and food content. This is consistent with the current trend in remote data collection from patients, as recently demonstrated during the COVID-19 pandemic [32]. This small study opens future avenues for clinical research about UPF consumption in children with obesity and the use of applications with pictures to collect nutritional intakes. Of note, our study was conducted prior to the COVID-19 pandemic and cannot thus address the psychosocial challenges of youth during the pandemic [33].

### Conclusions

In our study, the small group of adolescents had unbalanced eating habits despite being in a treatment program. They all defined their parent as being restrictive in terms of diet, and no parent reported a permissive food educational style. The consumption of UPFs was lower among adolescents whose parent was more restrictive, suggesting that adolescents have fewer opportunities to eat when some degree of restriction is applied by their parent. The parent’s food educational style and food choices available at home, including UPFs, may be a key target for personalized nutritional interventions in adolescents with obesity.

## Abbreviations

BMI: body mass index
PI: principal investigator
SNS: Swiss Nutrition Society
UPF: ultraprocessed food
